# Attention Enhancement for Exoskeleton-Assisted Hand Rehabilitation Using Fingertip Haptic Stimulation

**DOI:** 10.3389/frobt.2021.602091

**Published:** 2021-05-21

**Authors:** Min Li, Jiazhou Chen, Guoying He, Lei Cui, Chaoyang Chen, Emanuele Lindo Secco, Wei Yao, Jun Xie, Guanghua Xu, Helge Wurdemann

**Affiliations:** ^1^ School of Mechanical Engineering, Xi’an Jiaotong University, Xi’an, China; ^2^ State Key Laboratory for Manufacturing Systems Engineering, Xi’an Jiaotong University, Xi’an, China; ^3^ School of Civil and Mechanical Engineering, Curtin University, Perth, WA, Australia; ^4^ Department of Biomedical Engineering, Wayne State University, Detroit, MI, United States; ^5^ School of Mathematics, Computer Science and Engineering, Liverpool Hope University, Liverpool, United Kingdom; ^6^ Department of Biomedical Engineering, University of Strathclyde, Glasgow, United Kingdom; ^7^ Department of Mechanical Engineering, University College London, London, United Kingdom

**Keywords:** haptic feedback, hand rehabilitation, fingertip haptic stimulation, pneumatic haptic actuator, robot-assisted hand rehabilitation, hand exoskeleton

## Abstract

Active enrollment in rehabilitation training yields better treatment outcomes. This paper introduces an exoskeleton-assisted hand rehabilitation system. It is the first attempt to combine fingertip cutaneous haptic stimulation with exoskeleton-assisted hand rehabilitation for training participation enhancement. For the first time, soft material 3D printing techniques are adopted to make soft pneumatic fingertip haptic feedback actuators to achieve cheaper and faster iterations of prototype designs with consistent quality. The fingertip haptic stimulation is synchronized with the motion of our hand exoskeleton. The contact force of the fingertips resulted from a virtual interaction with a glass of water was based on data collected from normal hand motions to grasp a glass of water. System characterization experiments were conducted and exoskeleton-assisted hand motion with and without the fingertip cutaneous haptic stimulation were compared in an experiment involving healthy human subjects. Users’ attention levels were monitored in the motion control process using a Brainlink EEG-recording device and software. The results of characterization experiments show that our created haptic actuators are lightweight (6.8 ± 0.23 g each with a PLA fixture and Velcro) and their performance is consistent and stable with small hysteresis. The user study experimental results show that participants had significantly higher attention levels with additional haptic stimulations compared to when only the exoskeleton was deployed; heavier stimulated grasping weight (a 300 g glass) was associated with significantly higher attention levels of the participants compared to when lighter stimulated grasping weight (a 150 g glass) was applied. We conclude that haptic stimulations increase the involvement level of human subjects during exoskeleton-assisted hand exercises. Potentially, the proposed exoskeleton-assisted hand rehabilitation with fingertip stimulation may better attract user’s attention during treatment.

## Introduction

Stroke is a common global health problem and a principal contributor to acquired disability (Murphy and Werring, 2020). Many stroke survivors suffer from hand motor dysfunctions. Their abilities to live independently are greatly affected since hand functions are essential for our daily life (Heo et al., 2012). Because of the complexity of hand functions and the much larger area of cortex in correspondence with the hand than other limb parts, hand motion dysfunction is more challenging to recover than other limb parts (Yue et al., 2017) demanding research of hand motor recovery.

Hand rehabilitation requires continuous passive motion (CPM) exercises, which involve passive, repetitive tasks such as grasping, to provide motor sensory stimulation improving hand strength, range of motion, and motion accuracy with assistance from therapist or robotic assistive devices (Ueki et al., 2012). High costs of conventional treatments often prevent patients from spending enough time on necessary rehabilitation (Maciejasz et al., 2014). Virtual Reality (VR)-mediated motor interventions and robotic rehabilitation devices have now been introduced to address these shortcomings (Yue et al., 2017). VR allows patients to interact with simulated environments and perceive real-time performance feedback (Cho et al., 2014). A robotic rehabilitation device can act as an effective “therapist” that 1) delivers reproducible motor learning experiences, 2) quantitatively monitors patient performance, 3) adjusts rehabilitation training according to patients’ progress, and 4) ensures consistency in planning a therapy program (Henderson et al., 2008; Cho et al., 2014).

Robot-assisted rehabilitation has been proved to be effective in hand motor function improvements (Kutner et al., 2010; Carmeli et al., 2011). During the past few years, hand exoskeleton devices have drawn increasing research attention with promising results for hand rehabilitation (Haghshenas-Jaryani et al., 2017; Yap et al., 2017; Hadi et al., 2018; Li et al., 2019). Exoskeleton robots have many advantages such as portability, which have become the development trend of hand rehabilitation robots for stroke survivors (Yue et al., 2017). In this context, in our previous study, we proposed a hand exoskeleton that can assist both extension and flexion of fingers in CPM for hand rehabilitation purposes using a rigid-soft combined mechanism (Li et al., 2019).

Active enrollment in rehabilitation training yields better treatment outcomes (Ang and Guan, 2013; Teo and Chew, 2014). However, since the CPM training is passive, it is difficult for the patient to stay focused during the training process. Multi-mode sensory feedback during rehabilitation training can enrich experience to improve training involvement, enhance motor learning, help rebuilding the sensorimotor loop, and thus promote functional recovery of patients’ limbs (Sigrist et al., 2013; Takeuchi and Izumi, 2013; Sharififar et al., 2018). There have been several reports of rehabilitation training combining *visual* and/or *auditory* cues or stimuli (Secoli et al., 2011; Cameirão et al., 2012; Yue et al., 2017; Li et al., 2018). Tracking the user’s hand and providing task-specific visual feedback during rehabilitation training can increase the patient's engagement and motivation (Pereira et al., 2020). Auditory stimulation is helpful for rhythmic movements and improving exercise duration (Song and Ryu, 2016; Lee et al., 2018).

Stroke survivors with hand dysfunction may also lose part of *haptic sensation* in their hands (Heo et al., 2012). Haptic feedback can provide more sensation cues in virtual world during VR-mediated rehabilitation training, subsequently leading to improved motor relearning (Piggott et al., 2016). Hand exoskeleton can provide movement assistance to the hand during a CPM training process creating sensorimotor feedback. Cutaneous (also can be referred as tactile) inputs are generated by stimulating mechanoreceptors in the skin, and detect skin contact with objects and perception of surface properties (Lim et al., 2014). Combining cutaneous haptic stimulation to the fingertips with exoskeleton-assisted hand rehabilitation can provide sensorimotor and cutaneous haptic feedback simultaneously and may have potential to improve training involvement of stroke patients and thus promote the restoration of motor function. To the best of our knowledge, cutaneous haptic stimulation integrated with exoskeleton-assisted hand rehabilitation has not yet been reported.

Combining haptics with exoskeleton-assisted hand rehabilitation requires devices to provide compelling haptic sensations and, at the same time, be small, lightweight, inexpensive, and comfortable to wear. Since fingertips are more sensitive and tend to be involved in more contact interactions than other areas of hands, it would be most effective for cutaneous haptic devices to provide tactile sensation to fingertips rather than to the whole hand reducing the size and weight of any haptic feedback system. Due to the challenges of being small size and less complexity, wearable fingertip cutaneous haptic feedback systems have only started to be developed in recent years (Minamizawa et al., 2010; Pacchierotti et al., 2017; Schorr and Okamura, 2017; Zhai et al., 2020). Advances in soft robotics have provided a unique approach for conveying haptic feedback to a user by soft wearable devices. In our previous study, we created pneumatic haptic feedback actuators for multi-fingered palpation (Li et al., 2014a; Li et al., 2014b). Those actuators were fabricated *via* casting and molding using materials such as PDMS and silicone rubber. Such methods are expensive to replicate given the need to recreate a mold for every prototype iteration and the prototype quality is hard to control. In recent years, there has been a significant trend toward the use of 3D printing technology to fabricate soft material structures for soft robotic systems (Gul et al., 2018). The recent progress in soft material 3D printing techniques that allow cheaper and faster iterations of prototype designs have not been adopted to make haptic feedback actuators (Yap et al., 2016; Ang and Yeow, 2017; Yap et al., 2017; Gul et al., 2018).

This paper builds on our previous research investigating a rigid-soft combined mechanism for a hand exoskeleton that can assist both extension and flexion of fingers in hand rehabilitation (Li et al., 2019). Here, we presents the creation and validation of a fingertip cutaneous haptic stimulation system for exoskeleton-assisted hand rehabilitation using 3D-printed pneumatic actuators to improve training involvement of stroke patients and promote motor function recovery. The proposed fingertip cutaneous haptic stimulation is integrated with the hand exoskeleton to form a hand rehabilitation system. By combining the sensorimotor feedback created by exoskeleton-assisted hand movements and the cutaneous haptic feedback generated by the fingertip cutaneous haptic stimulation, the exoskeleton-assisted hand CPM exercise becomes more attention-catching making the patients focus more on the process of hand extension and flexion training.

*Hand Rehabilitation System* describes the system design. *Experiment of Normal Contact Force Change Pattern During Glass Grasping* shows the experiment to investigate the change pattern of the fingertip contact forces during the process of grasping a glass to establish a glass-grasping model for fingertip cutaneous haptic stimulation. *System Performance Validation and Influence of Haptic Stimulation on User’s Attention* provides the system characterization and user study. The experimental results are analyzed in *Results*. Discussions are provided in *Discussions*.

## Materials and Methods

### Hand Rehabilitation System

#### Concept of Combining Hand Exoskeleton and Fingertip Cutaneous Haptic Stimulation

**FIGURE 1:**
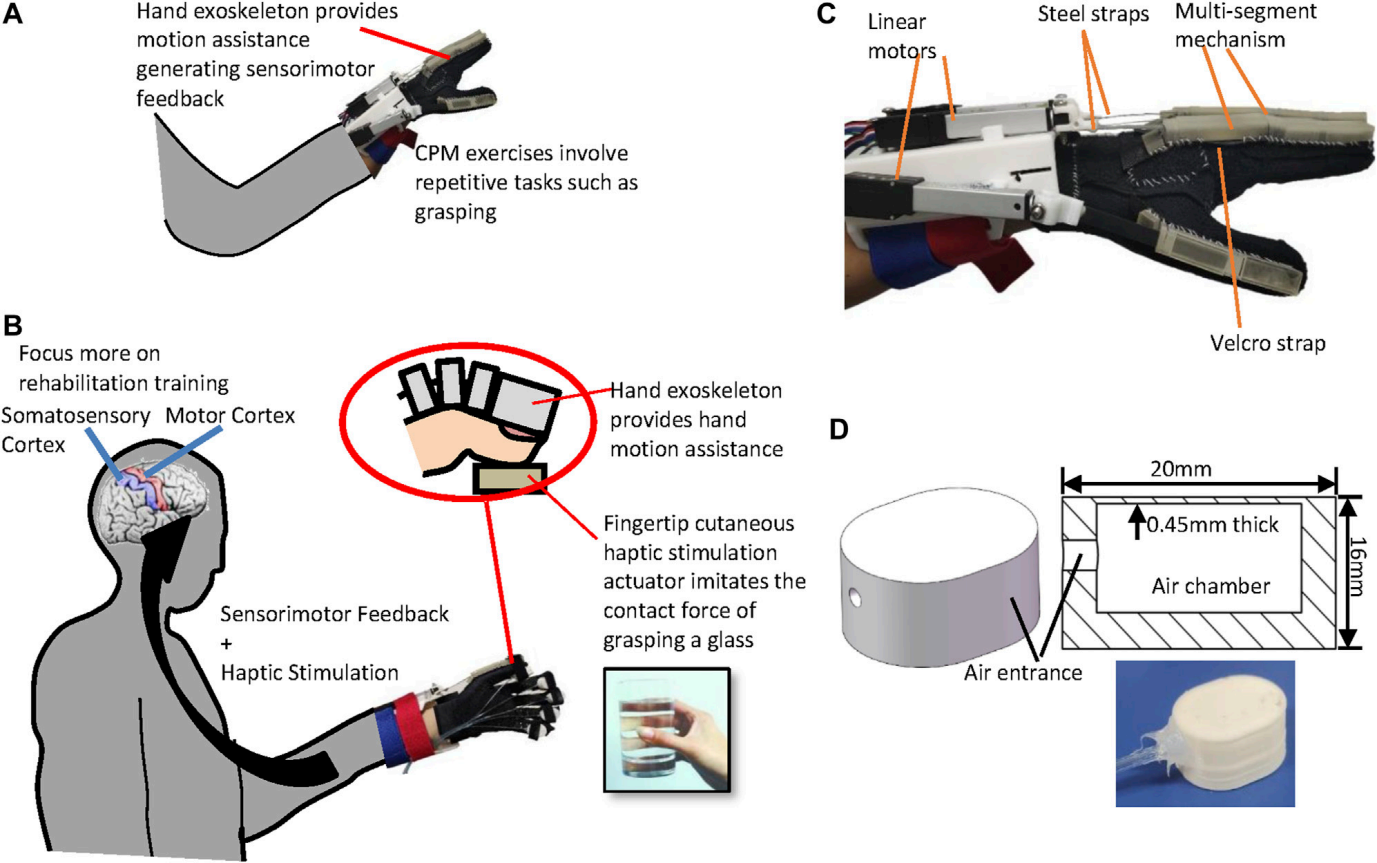
Illustrations of **(A)** the conventional exoskeleton-assisted hand rehabilitation, **(B)** our hand rehabilitation robot system combining hand exoskeleton and fingertip haptic stimulation, **(C)** hand exoskeleton, and **(D)** the proposed pneumatic haptic stimulation actuator.

Figure 1A shows the conventional exoskeleton-assisted hand rehabilitation and Figure 1B presents the concept of our hand rehabilitation system combining a hand exoskeleton with fingertip cutaneous haptic stimulation. In the conventional exoskeleton-assisted hand rehabilitation, only a hand exoskeleton is used to provide extension and flexion assistance to the patient’s fingers during a CPM training. This passive, repetitive exercises can provide sensorimotor feedback to the patient to improve hand functions in terms of range of motion and strength. However, since the training is passive, it is difficult for the patient to stay focused. Therefore, we proposed to add haptic feedback to the fingertips to improve the patient’s involvement in the exoskeleton-assisted rehabilitation training process. The hand exoskeleton - driven by linear motors - supports human fingers to conduct flexion and extension motions resulting in sensorimotor feedback. During the process, haptic stimulation actuators - mounted on the fingertips - generate contact forces between the actuators and the fingertips enhancing patient’s somatosensory stimulation. Integrating haptic stimulation with exoskeleton-assisted hand rehabilitation aims to improve the patient’s involvement in the training process 1) enhancing motor learning, 2) helping the recovery of sensorimotor feedback loop, and 3) promoting the recovery of hand motor function.

#### Hand Exoskeleton

In our previous study, we proposed a hand exoskeleton that can assist both the extension and flexion of the fingers using a rigid-soft combined mechanism (Li et al., 2019). Please note that the hand exoskeleton was not used to provide kinesthetic feedback of the interaction between the fingers and the virtual objects [like the haptic exoskeletons in (Secco and Maereg, 2019; Wang et al., 2020)] but to provide movement assistance to the hand. Each finger is driven by one actuator containing a linear motor, a steel strap, and a multi-segment mechanism (see Figure 1C). Each segment of the mechanism is made of VisiJet Crystal material using a rapid prototyping machine (3D Systems MJP3600). Five finger actuators are attached to a fabric glove *via* Velcro straps. Linear motors are attached to a rigid part, which are fixed to the forearm by a Velcro strap. Each steel strap are attached to a motor by a small rigid 3D-printed part. The rigid part are made of PLA using a rapid prototyping machine (D3020, Shenzhen Sundystar technology co. Ltd., China). The spring layer bends and slides when it is pushed by the linear motor. The multi-segment structure then becomes like a circular sector. The spring layer is straightened when pulled by the linear motor. The linear motors (L12-50-210-12-I, Firgelli Technologies, Ferndale, MI, United States) allow a stroke up to 50 mm, with a maximum speed of 5 mm/s, and a maximum force of 30 N. The weight of the overall device is 435 g, including the glove, the multi-segment mechanism, and the motors.

#### 3D-Printed Fingertip Cutaneous Haptic Stimulation Actuators

Researchers used actuators with air cambers and inflatable surfaces to create the contact force between the fingertip and the actuator surface for fingertip cutaneous haptic feedback (Li et al., 2014a; Li et al., 2014b; Lim et al., 2014; Sarkar et al., 2020). Casting and molding fabrication methods were used to create such actuators with materials such as PDMS and silicone rubber (Li et al., 2014a; Li et al., 2014b; Lim et al., 2014; Sarkar et al., 2020). However, such methods are expensive to replicate given the need to recreate a mold for every prototype iteration and the prototype quality is hard to control. To solve this problem, we adopted soft material 3D printing techniques which allow cheaper and faster iterations of prototype designs to make soft pneumatic fingertip cutaneous haptic feedback actuators in this study. As shown in Figure 1D, the novel proposed haptic stimulation actuators contains an air chamber surrounded by a 0.45 mm thick working surface, a 2.5 mm thick bottom and a 2.5 mm thick oval side. The actuator was 3D printed using a Ninjaflex soft material (NinjaTek, 2019): a 3D printer model Lulzbot TAZ 6 with a resolution of 0.15 mm was used. No support materials were required to print the chambers. When printing the parts above the chambers, the material sagged a little for the first few layers without affecting the function of the actuators. An air tubing with a diameter of 2 mm is connected to the actuator by using RTV 108 clear silicone rubber adhesive sealant (Momentive, 2020). When air is injected into the air chamber, the working surface inflates increasing the contact force between the actuator and the user’s fingertip while the bottom and side shows a little deformation. The relation between the input pressure and the contact force on the actuator surface is determined through a calibration set of experiments as it is shown in *Haptic Actuator and Haptic Stimulation System Characterization* and *Experimental Results of Characterization*. An actuator fixture with 3D-printed PLA part and Velcro was used to attach the haptic actuator to the user’s fingertip.

#### Fingertip Cutaneous Haptic Stimulation

The hand exoskeleton is controlled to drag the user’s hand conducting the motion of grasping. During the flexion and extension motion of the exoskeleton, the haptic stimulation force varies to simulate the contact force when the hand interacts with a virtual object (e.g., a glass in our case). According to the design of the hand exoskeleton, the change of motor travel distance and the bending angle of the exoskeleton fingers have a linear relation (Li et al., 2019). The motor travel distance is monitored through the motor stroke feedback signal which is acquired by using an analog input/output module (JY-DAM10AIAO, Beijing Elit Gathering Electron, Beijing, China). The finger joint angles are then acquired through the motor stroke data. When the finger is about to touch the simulated glass, then the haptic feedback actuator is activated. The corresponding target contact force for each fingertip is calculated through a glass-grasping model, which is established based on the data from the experiment shown in *Experiment of Normal Contact Force Change Pattern During Glass Grasping* and *Typical Normal Contact Force Change Pattern*. The required pressure is calculated according to the target contact force by using the experimentally determined relation between the input pressure and the contact force on the actuator surface expressed in Eq. 1 and shown in *Experimental Results of Characterization*. The corresponding analog signal is then transmitted to a pressure regulator (SMC ITV0010, Japan) through the analog output module. Pressurized air is provided by an air compressor (U-STAR601, U-STAR, China).

#### System Integration and Control

**FIGURE 2:**
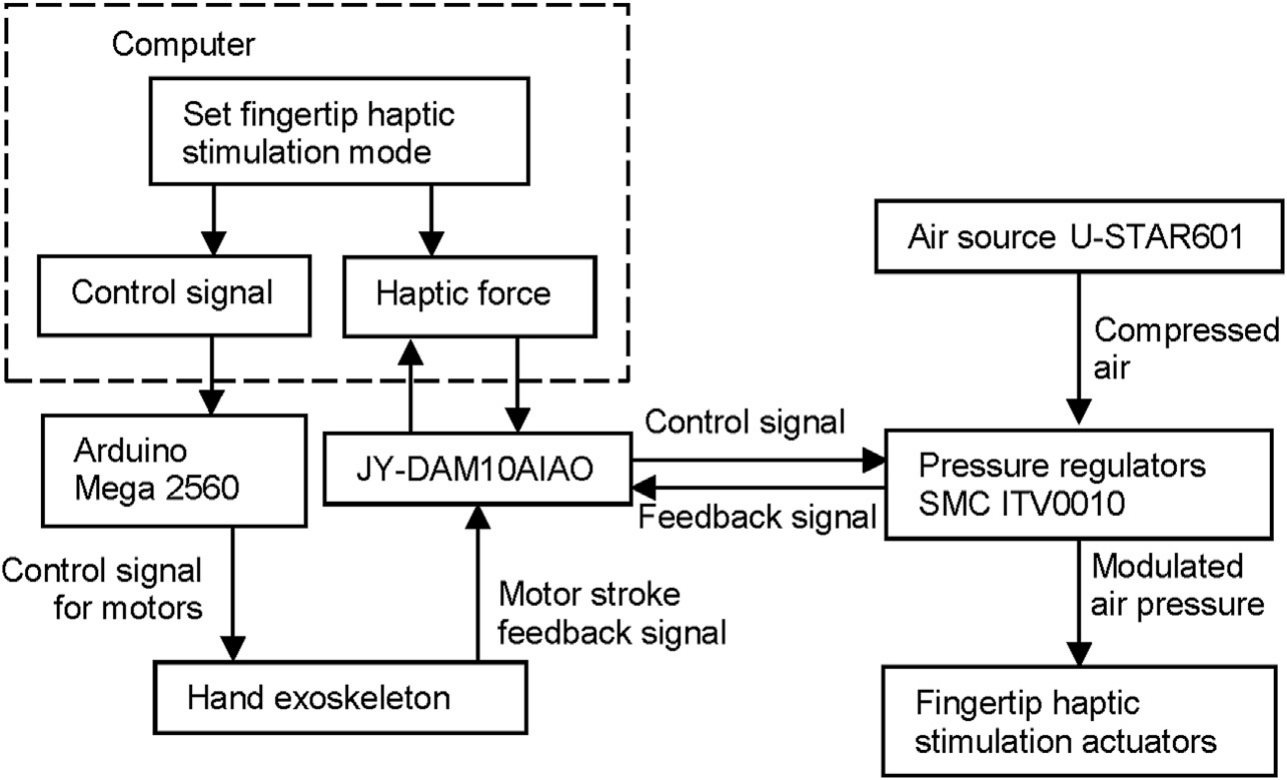
System integration and control of the hand rehabilitation combining the hand exoskeleton (left diagram) and the fingertip haptic stimulation (right diagram).

Figure 2 shows the overall system integration and control of the hand rehabilitation system, combining the exoskeleton-assisted hand motion and the fingertip cutaneous haptic stimulation. The motor stroke sequence is embedded in an Arduino Mega 2560. When the computer sends a start command, the Arduino Mega 2560 starts to send the control signals to the linear motors in the hand exoskeleton. The motor stroke feedback signals are sent to an analog input/output module JY-DAM10AIAO. The target haptic force is calculated, according to the selected mode and the feedback motor stroke information and transfers to the analog input/output module JY-DAM10AIAO to control the air pressure inside the fingertip cutaneous haptic stimulation actuators *via* the pressure regulators SMC ITV0010. Pressurized air is provided by an air compressor U-STAR601 as reported before. The feedback signals from the pressure regulators are monitored by the JY-DAM10AIAO device.

### Experiment of Normal Contact Force Change Pattern During Glass Grasping

An experiment was conducted to investigate the change pattern of the fingertip contact forces during the process of grasping a glass. A glass-grasping model for fingertip cutaneous haptic stimulation can then be established based on the contact force change pattern during glass grasping.

**FIGURE 3:**
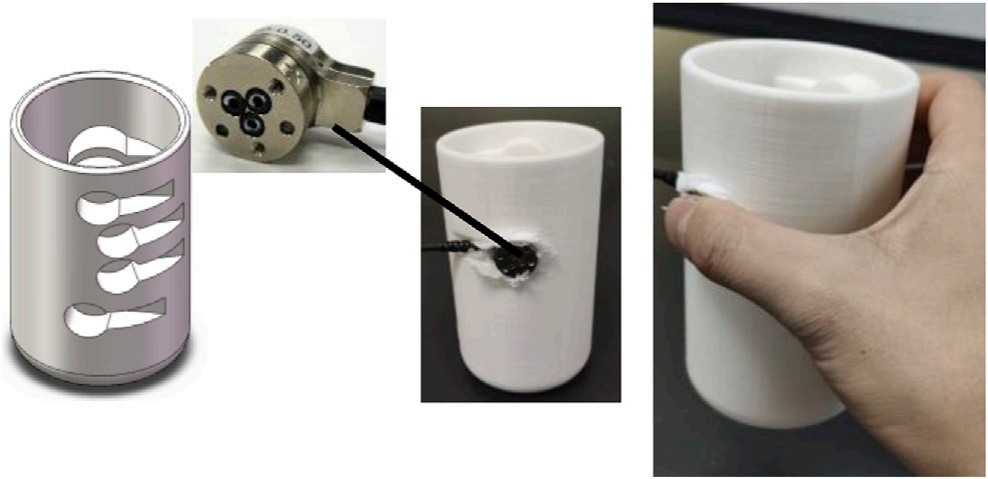
A 3D-printed glass-shaped object with force sensor embedded for the experiment investigating the change pattern of fingertip contact forces during the process of grasping a glass.

Ten participants (7 males and 3 females with an average age of 27, all right-handed) were involved in this experiment. As shown in Figure 3, a 3D-printed glass-shaped object (diameter: 70 mm, height: 120 mm, net weight: 150 g) that could embed force sensors was applied. The material of this object is PLA. The 3D-printed glass-shaped object contains 5 grooves to install force sensors (SI-12-0.12, ATI Nano 17, United States) corresponding to the five fingers. Tissue is used to fill the gap between the groove and the sensor in order to secure the sensor. The weight of the glass was changed by adding water into the glass. The weight of the tested glass was 150 g, 200 g, 250 g, and 300 g, respectively. During the test, the participants were required to use the same grasping pattern for the same weight of different trials. The test was repeated five times. This study with human participants was approved by the Institutional Review Board of Xi’an Jiaotong University. All subjects signed a written consent before the beginning of the experiments.

### System Performance Validation and Influence of Haptic Stimulation on User’s Attention

#### Haptic Actuator and Haptic Stimulation System Characterization

**FIGURE 4:**
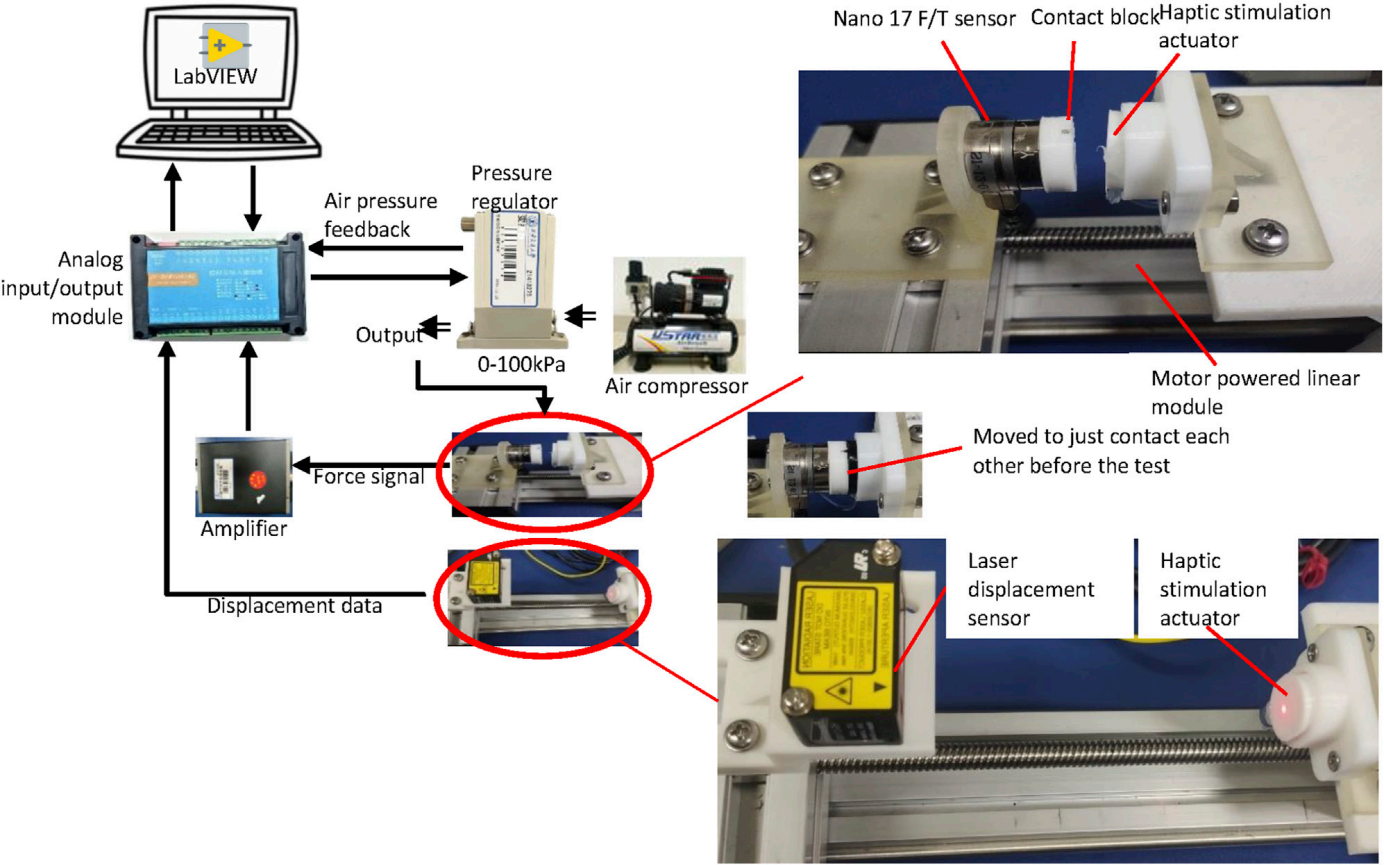
Experimental set-up for the deformation response and generated contact force of the actuator.

The weight of five haptic actuators was measured using an electronic scale (measurement range 0–100 g with a resolution of 0.01 g). The deformation response of the actuators was examined under different inflation pressures ranging from 0 to 100 kPa with an interval of 0.5 kPa. The deformation of the actuators was measured by using a laser displacement sensor (HG C1100, Panasonic, Japan, repeated accuracy 79 μm, measurement range ±35 mm, light spot diameter 120 μm) (see Figure 4). Five actuators were examined. An analog input/output module JY-DAM10AIAO was used to provide the control signal to the pressure regulator SMC ITV0010. The pressure regulator reduced the air pressure from the air source and inflated the actuator with an amount of pressure which is proportional to the given control signal.

As shown in Figure 4, the generated contact force was also calibrated when the actuators were inflated and deflated between 0 and 100 kPa for five times. One inflation and deflation process lasted 100 s. A haptic stimulation actuator was fixed at one side of a guide rail. An ATI Nano 17 Force/Torque sensor SI-12-0.12, which was attached to a contact block printed using Ninjaflex for force measurement, was fixed to the sliding block on the guide rail. Before the test, they were moved to just contact each other. Twelve actuators were examined.

**FIGURE 5:**
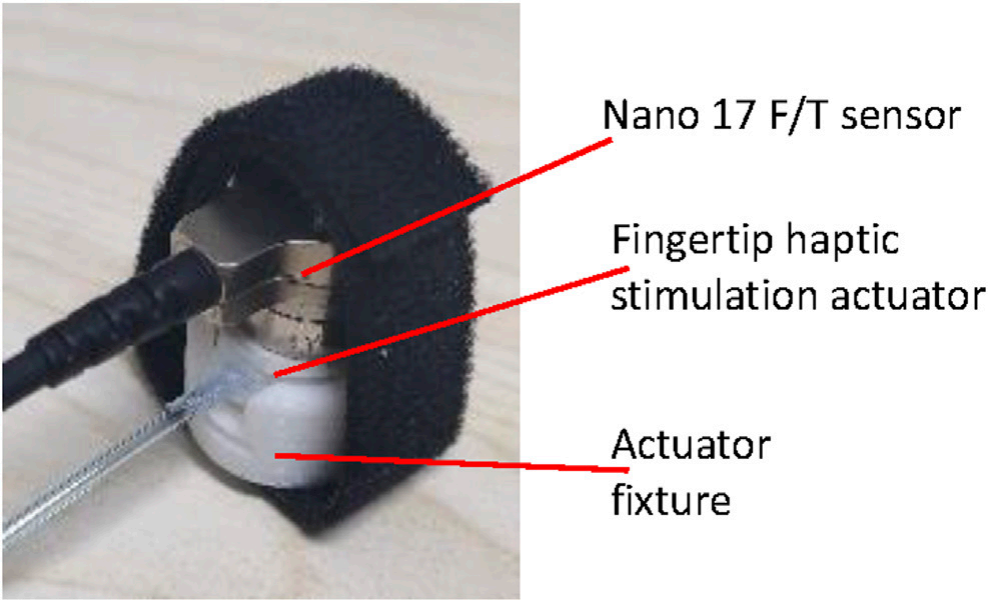
Experimental set-up for the system response characterization.

The response time of the haptic stimulation system was also examined. The haptic stimulation system was controlled to generate stimulation force from 0 to 4 N and then back to 0 N. As shown in Figure 5, a Force/Torque sensor ATI Nano 17 SI-12-0.12 was used to replace the fingertip and capture the contact force. The experiment was repeated for three times.

#### Experimental Protocol of User Study

**FIGURE 6:**
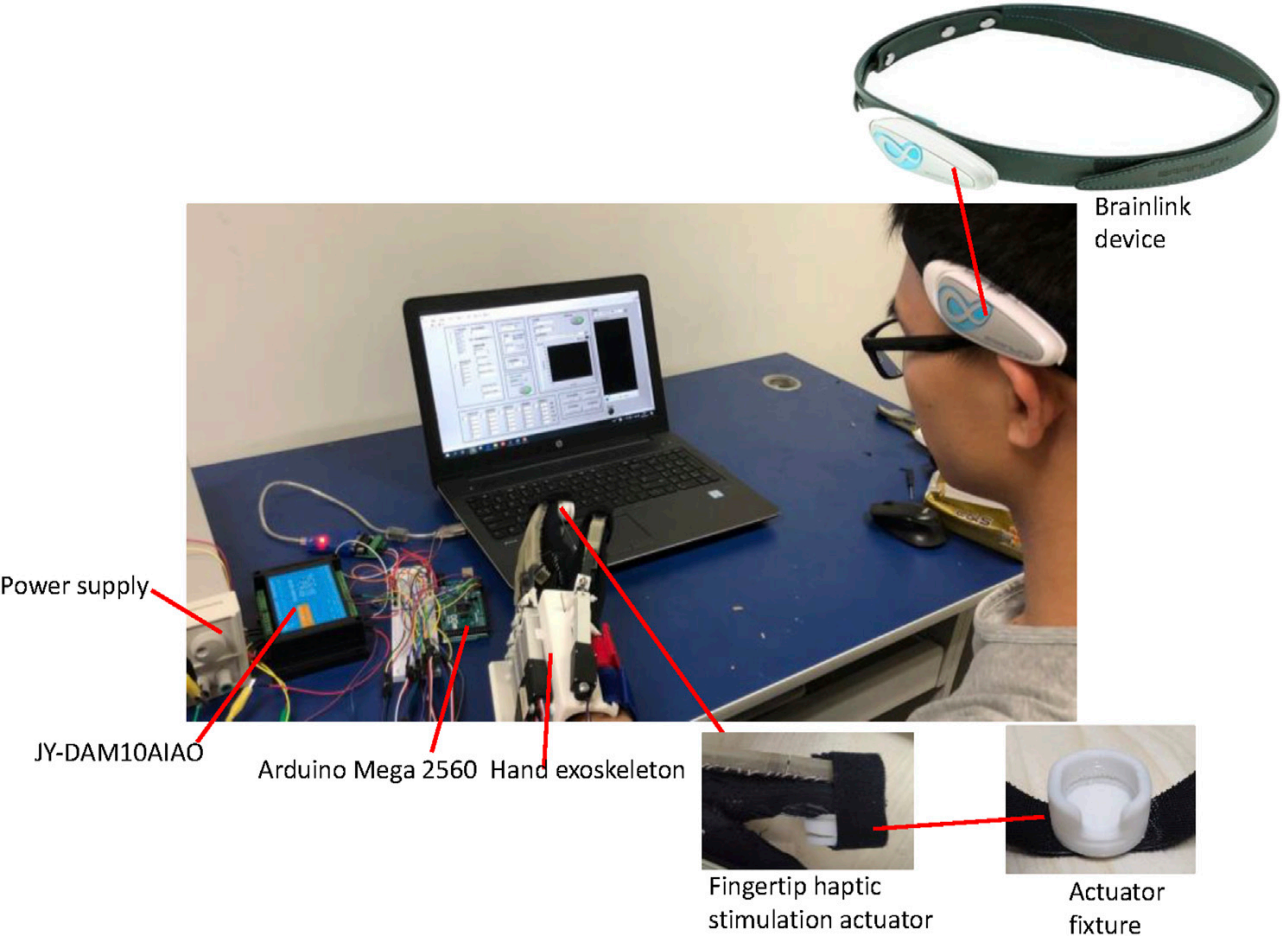
Experimental set-up for the user study.

In this study, we assumed that adding fingertip cutaneous haptic feedback to exoskeleton-assisted hand extension and flexion motions for rehabilitation purposes could improve the participation of the user in the rehabilitation training process. A user study was conducted to investigate this attention enhancement effect of integrating haptic stimulation into the exoskeleton-assisted hand rehabilitation. The experimental set-up is shown in Figure 6. During the experiment, the participants’ attention levels were monitored in real time by using a Brainlink Lite device. Brainlink is a commercial, easy-to-wear, inexpensive EEG detection device that consists of three dry electrodes, including an EEG signal channel, a reference electrode, and a grounding electrode. The Brainlink sampling rate is 512 Hz with a frequency range of 3–100 Hz. This device records EEG the band power values of the delta, theta, alpha, beta, and gamma waves. A ThinkGear AM (TGAM) module (NeuroSky, Inc., Silicon Valley, United States) was used to process the brain signals. The outputs of this module report the attention and relaxation of the user brain *via* a built-in patented eSense biometric algorithms which measure whether the brain is focused or relaxed (NeuroSky, 2018). The parameter (i.e., the Attention and the so called Meditation) are calculated in a range between 1 and 100. Thus, the current attention level of the subject was recorded through the BrainLink, in order to analyze whether the subject was focused on the rehabilitation process during our experiments.

The development of rehabilitation robots usually consists of several stages. Validating rehabilitation robots with healthy participants is a common practice in the early stages of development (Chisholm et al., 2014; Li et al., 2017; Becker et al., 2019; Nicholson-Smith et al., 2020). Therefore, in this preliminary study, thirteen healthy participants were involved in this user study to prove the attention enhancement effect of integrating haptic stimulation into the exoskeleton-assisted hand rehabilitation. Three experimental modes were examined including 1) grasping motion assisted by exoskeleton without haptic stimulation, 2) grasping motion assisted by exoskeleton with haptic stimulation (simulated glass weight of 150 g), and 3) grasping motion assisted by exoskeleton with haptic stimulation (simulated glass weight of 300 g).

Five cycles of the flexion/extension motion were involved in each trial. Four trials were conducted by each participant. The sequence of the five experiment parts was pseudo random. During the experiment, the attention levels were recorded at a sampling rate of 1 Hz. The study was approved by the Institutional Review Board of Xi’an Jiaotong University. All subjects signed a written consent before the start of the experiment.

#### Statistical Analysis

The primary outcome of interest in this study was the average change in intention level in different groups. A Shapiro-Wilk test was used to check the sample normality. A Levene test was used to examine the homogeneity of variance. One-way ANOVA with PostHoc LSD was used to determine the significant difference among those groups. A single-tailed pairwise student t-test was used to compare the attention level difference between every two modes. Since three experiment modes were compared in this multiple hypothesis testing, a Benjamini-Hochberg method was used to control the false discovery rate. For all analyses with *p* value smaller than 0.05 was considered statistically significant. All analyses were performed using R software (Version 3.6.3, The R Foundation).

## Results

### Typical Normal Contact Force Change Pattern

**FIGURE 7:**
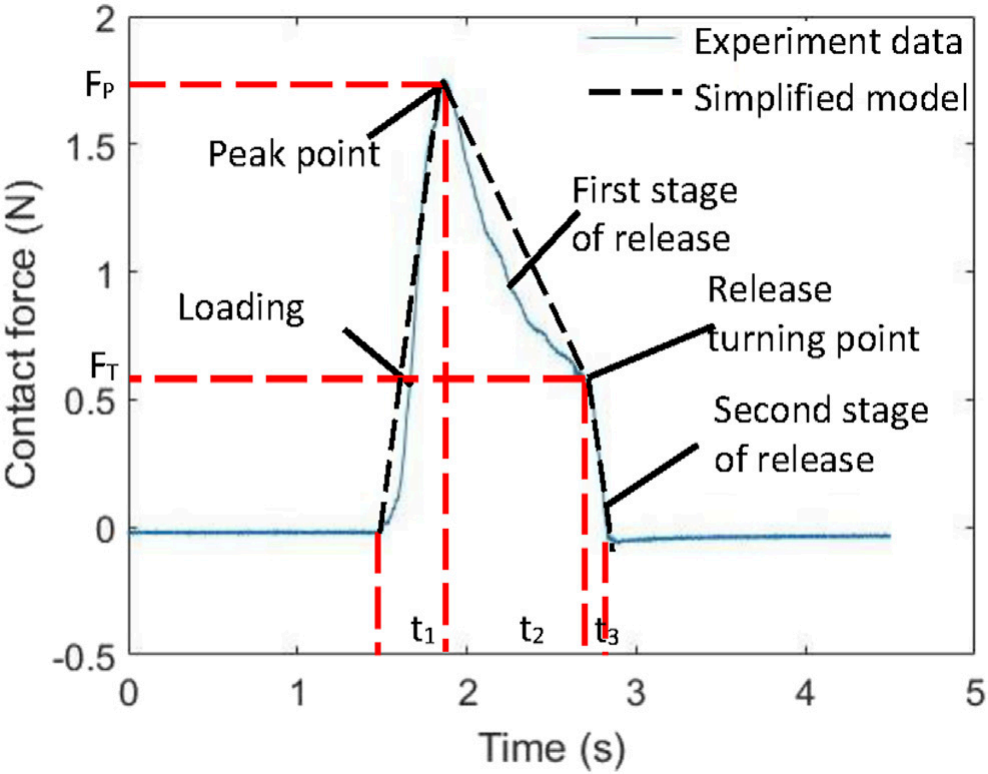
A typical normal contact force change pattern.

Figure 7 shows a typical normal contact force change pattern during the experiment. The data represents the middle finger contact force from a grasping trial of one of our experimental participants. Similar patterns can be observed in the other trials. In general, the process of grasping the glass can be divided into three stages: 1) the rapid loading stage, 2) the slow release stage and 3) the rapid release stage.

**FIGURE 8:**
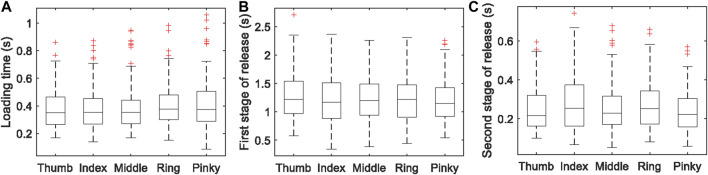
The time length of each stage of glass-grasping: **(A)** loading, **(B)** first stage of release, and **(C)** second stage of release. If the data are greater than *q*_3_ + 1.5 × (*q*_3_ – *q*_1_) or less than *q*_1_ – 1.5 × (*q*_3_ – *q*_1_), where *q*_1_ and *q*_3_ are 25th and 75th percentiles of the sample data, they are marked red.

**FIGURE 9:**
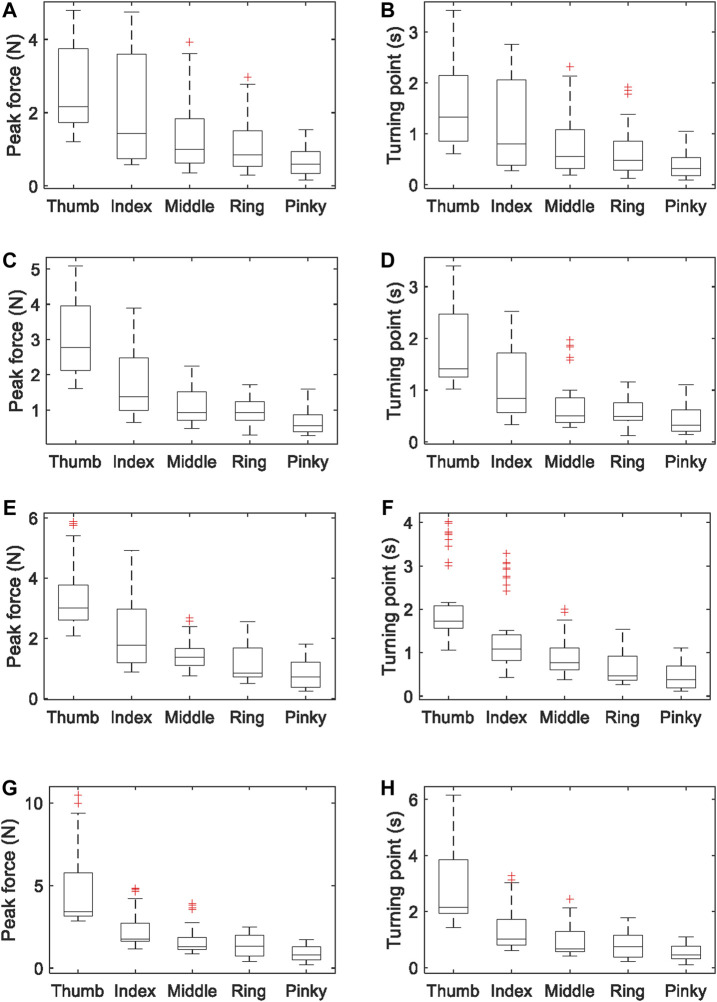
**(A)** Peak forces (mean ± SD) and **(B)** turning points when grasping a 150 g simulated glass of water; **(C)** peak forces and **(D)** turning points when grasping a 200 g simulated glass of water; **(E)** peak forces and **(F)** turning points when grasping a 250 g simulated glass of water; **(G)** peak forces and **(H)** turning points when grasping a 300 g simulated glass of water. If the data are greater than *q*_3_ + 1.5 × (*q*_3_ – *q*_1_) or less than *q*_1_ – 1.5 × (*q*_3_ – *q*_1_), where *q*_1_ and *q*_3_ are 25th and 75th percentiles of the sample data, they are marked red.

In order to determine the force curve of grasping the glass, five variables need to be defined: loading time *t*_1_, unloading time *t*_2_ in the first stage, unloading time *t*_3_ in the second stage, peak force *F*_P_, and unloading force node *F*_T_. Figure 8 shows the data of the time length of each stage. The data of duration in each of the three stages (*t*_1_, *t*_2_ and *t*_3_) shows individual differences, but the average stage duration of five fingers are consistent. Figure 9 shows the data of peak forces and release turning points. The thumb borne the maximum normal force when grasping the simulated glass of water. There is a trend of decreasing peak force and turning point force from the thumb to the little finger.

Therefore, in our glass-grasping model for fingertip haptic stimulation, the average stage duration of the five fingers is used as the stage duration of the haptic stimulation actuator. The loading stage *t*_1_, first stage of release *t*_2_, and the second stage of release *t*_3_ are 0.36, 1.20 and 0.24 s, respectively. Two weights of glass of water (150 and 300 g) were simulated. The average peak forces *F*_P_ and turning points *F*_T_ from the experiment were used in the model for fingertip haptic stimulation (see Table 1).

**TABLE 1:** Peak forces *F*_P_ and turning point forces *F*_T_ in our glass-grasping model for fingertip haptic stimulation.

	Peak force (N)		Turning point force (N)	
Item	150 g	300 g	150 g	300 g
Thumb	2.16	3.43	1.33	2.15
Index	1.44	1.76	0.80	1.03
Middle	1.00	1.28	0.55	0.68
Ring	0.85	1.34	0.48	0.75
Pinky	0.59	0.79	0.32	0.46

### Experimental Results of Characterization

**FIGURE 10:**
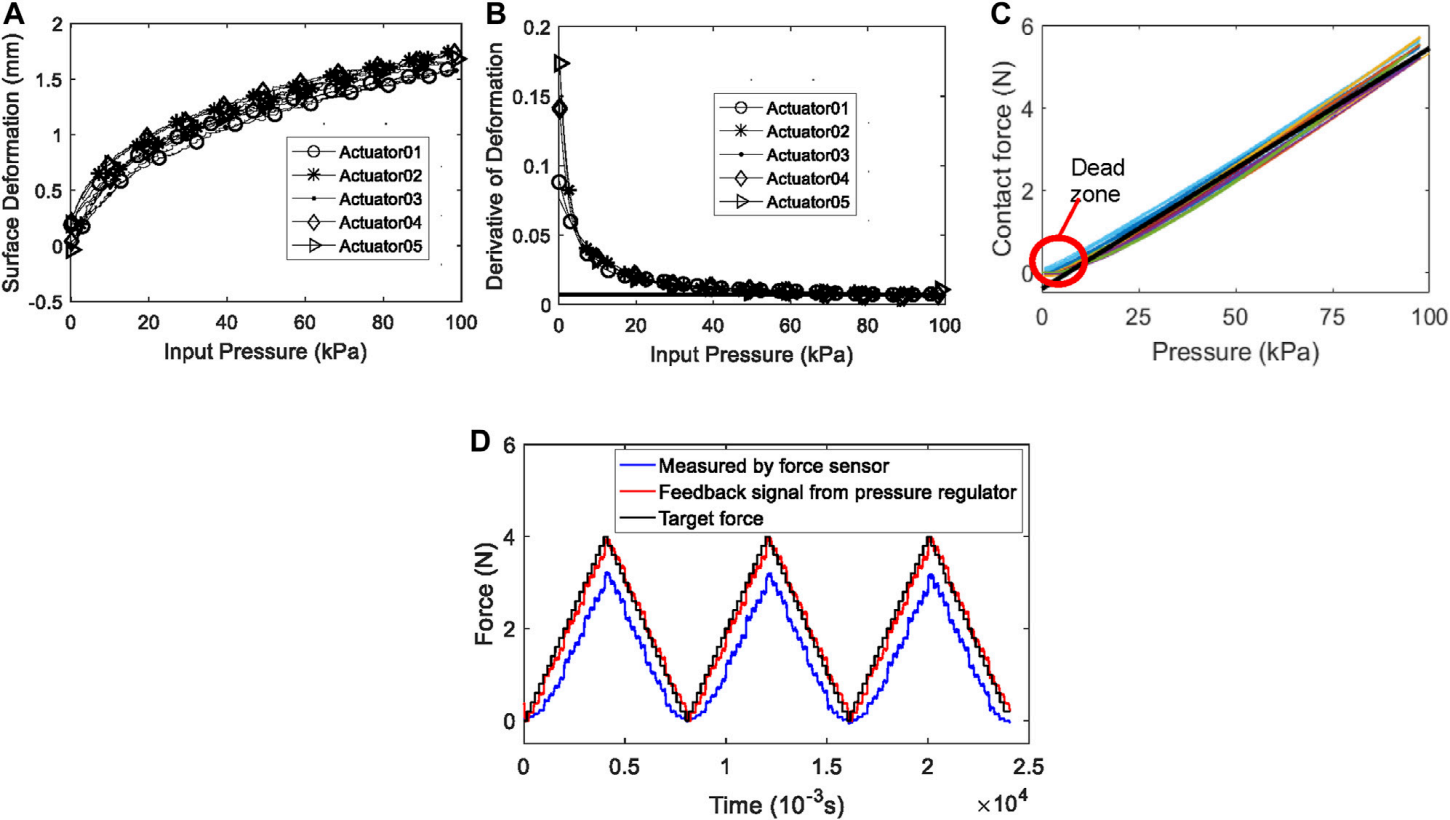
Characterization experimental results: **(A)** the relationship between surface displacement and input pressure, **(B)** the derivative of the surface displacement corresponding to the input pressure, **(C)** the relationship between the input pressure of the actuator and the contact force on the actuator surface, and **(D)** the measured force compared to the target force.

The weight of a haptic actuator is 2.5 ± 0.22 g. The haptic actuator with the actuator fixture weighted 6.8 ± 0.23 g. As shown in Figure 10A, the surface displacements of the haptic actuators are nonlinear in the low pressure range (0–40 kPa), whereas in the high pressure range (40–100 kPa), they present a good linear feature. The curve shown in Figure 10B was obtained by taking the derivative of the surface displacement with respect to the input pressure. The derivative of the actuator at the input pressure of nearly 100 kPa is close to a constant of 0.007 (as reported in black line within the figure), and there is no obvious abrupt change. Therefore, the maximum output pressure of the pneumatic proportional valve (100 kPa) did not exceed the upper limit of the actuator.

As shown in Figure 10C, the differences of the force output distribution among the actuators are negligible. There is an approximate linear relation between the contact force on the actuator surface and the input air pressure. Therefore, the fitting relation between the input pressure and the contact force on the actuator surface was acquired with linear least square fitting using the data from all the 12 actuators. This relation can be expressed as

F=0.0585P−0.4055, (1)

where *F* is the generated contact force with the unit of N; *p* is the input pressure with the unit of kPa. The test results show that the maximum output force of the 3D-printed pneumatic haptic stimulation actuator was 5.436 ± 0.171 N. Almost all of the actuators have a dead zone in the low-pressure range. The mean dead zone pressure of the 12 actuators is 4.233 kPa. Therefore, the actuators should be pre-inflated with about 5 kPa before using it. In general, the performance of the produced actuators is consistent and stable. The hysteresis negligible.

Figure 10D shows the measured force compared to the target force during the system response experiment. The average response time of the haptic stimulation system to the input control signal is 0.17 s. The ratio of the output force of the actuator as it was monitored by the force sensor to the target output force is 79.5%. 20.5% of the output force is converted into the elastic deformation of the Velcro. This loss of the output force is taken into account by compensating the input signal.

### Experimental Results of User Study

**FIGURE 11:**
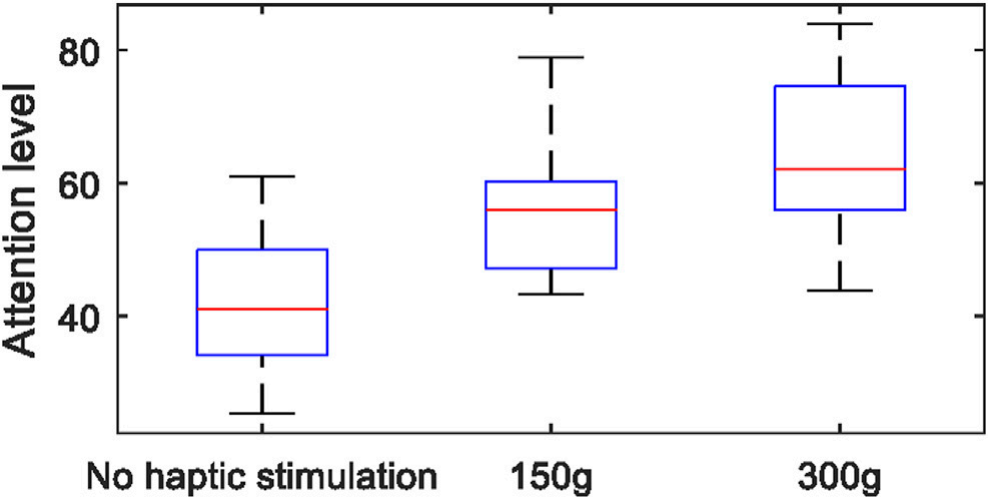
The attention levels of the participants during the experiment.

An average attention level was calculated for each trial. There were 52 attention level values (4 trials × 13 participants) for each mode. The average attention level of each experiment mode fits a normal distribution (Shapiro-Wilk test, *p* > 0.05), (in exoskeleton only group: W = 0.9748, *p* = 0.3323; in 150 g glass group: W = 0.9650, *p* = 0.1291, in 300 g glass group: W = 0.9734, *p* = 0.2932). The Levene test confirmed the homogeneity of variance (*p* = 0.154). As shown in Figure 11, the average attention level for those three experiment modes was 47.5 ± 12.34 (Mean ± SD), 56.1 ± 9.27, 63.6 ± 10.08, respectively. There was a significant difference between groups (One Way ANOVA, PostHoc LSD, *p* < 0.05). As shown in Table 2, participants had significantly higher attention levels in haptic stimulation than the group that only exoskeleton was used to drag the fingers (Paired *t* test, *p* = 0.000); participants have significantly higher attention levels in the higher stimulation level group (simulating grasping a 300 g glass) than the lower stimulation level group (simulating grasping a 150 g glass) (Paired *t* test, *p* = 0.000).

**TABLE 2:** The results of student t-tests with Benjamini-Hochberg correction.

Item	*p*
Exoskeleton only vs*.* Haptic stimulation simulating 150 g glass	3.820 × 10^−5**^
Exoskeleton only vs*.* Haptic stimulation simulating 300 g glass	1.724 × 10^−9**^
Haptic stimulation simulating 150 g glass vs*.* Haptic stimulation simulating 300 g glass	5.515 × 10^−6**^

^**^Stronger significance than at the 1% level.

^*^Significance at the 5% level.

## Discussions

This paper presents a hand rehabilitation system with the functions of exoskeleton-assisted hand movements and fingertip haptic stimulation to improve training involvement of stroke patients and promote the rehabilitation of motor function. The hand rehabilitation system is consisted of a fingertip haptic stimulation system with soft material 3D-printed pneumatic actuators, a hand exoskeleton using a rigid-soft combined mechanism, and a fingertip stimulation method imitating the contact force of grasping a glass during exoskeleton-assisted glass-grasping motion. The main contributions of this paper include 1) combining cutaneous haptic stimulation to the fingertips with exoskeleton-assisted hand rehabilitation to provide sensorimotor and cutaneous haptic feedback simultaneously; 2) adopting soft material 3D printing techniques to make soft pneumatic fingertip haptic feedback actuators achieving cheaper and faster iterations of prototype designs with consistent quality; 3) experimentally verifying the assumption that adding fingertip cutaneous haptic stimulation to exoskeleton-assisted hand extension and flexion motions can improve the training involvement of the user.

According to Pacchierotti et al. (2017), the average weight of the eighteen reviewed wearable haptic devices for the fingertip is 31.4 g (at the fingertip) and the smallest dimensions of the twenty reviewed wearable haptic devices for the fingertip is 12 × 12 × 30. The proposed 3D-printed pneumatic haptic stimulation actuator is small (16 × 16 × 20), wearable, and light-weight (6.8 ± 0.23 g each with a PLA fixture and Velcro). The maximum continuous normal force the proposed fingertip haptic device can generate is around 5.4 N while this figure of other wearable haptic devices ranges from 1.5 to 6.72 N (Sarakoglou et al., 2012; Prattichizzo et al., 2013; Chinello et al., 2015; Girard et al., 2016). As shown in Figure 10, the performance of the produced actuators is consistent and stable with small hysteresis. The current fabrication process limited the further miniaturization of the actuator (Maereg et al., 2017). During the 3D printing process, the working surface of the haptic feedback actuator was facing down to ensure the quality of this surface. Since no support materials were used to print the chamber, the bottom of the actuator (facing up during printing) would sag for the first few layers when printing. In order to ensure that the sagging material does not touch the working surface and has very little influence on the performance of the haptic actuator, a thick air chamber is required. What’s more, the bottom surface should not deform too much when the actuator is activated. Therefore, the bottom surface of the actuator is required to be much thicker than the working surface. To further improve the fabrication process and miniaturize the actuator, further study is required. Moreover, the output contact force of the actuator was not monitored in the current system. Therefore supplementary work is required to improve the fabrication process, to miniaturize the actuator, to generate the tangential contact force, and to improve the actuator’s control. Building a prosthetic hand with haptic feedback is an emerging research trend (Raspopovic et al., 2014). In this study, our hand exoskeleton and the fingertip haptic feedback system are designed for stroke rehabilitation, but the proposed haptic stimulation system may also have potential to be used for restoring tactile sensory feedback in hand prostheses. Clinical studies will be performed in the future.

In the conventional exoskeleton-assisted hand rehabilitation process, only a hand exoskeleton is used to provide extension and flexion assistance to the patient’s fingers during a CPM training. The passive repetitive exercises can provide sensorimotor feedback to the patient. However, since the training is passive, it is difficult for the patient to stay focused. Therefore, we proposed to add haptic feedback to the fingertips to improve the participation of the patient (indicated by the attention level) in the exoskeleton-assisted rehabilitation training process. To the best of our knowledge, cutaneous haptic stimulation integrated with exoskeleton-assisted hand rehabilitation has not yet been reported, other than in our study. We assumed that adding fingertip cutaneous haptic feedback to exoskeleton-assisted hand extension and flexion motions for rehabilitation purposes could improve the participation of the user in the rehabilitation training process. To verify this assumption, exoskeleton-assisted hand trainings with and without haptic stimulation were compared in an experiment involving healthy human subjects in this study. The experiment of the user study showed that participants had significantly higher attention levels when fingertip cutaneous haptic stimulations were added compared to when only the exoskeleton was used to drag the fingers (*p* = 3.820 × 10^−5^, *p* = 1.724 × 10^−9^). This result confirms that adding haptic stimulation to exoskeleton-assisted hand movements significantly increase the attention levels of the participants. The increased attention levels of the participants may suggest the increase of the subjects’ active involvement during the exoskeleton-assisted motion training process. Further, the increased active involvement of the subjects may lead to better training outcomes (Ang and Guan, 2013; Teo and Chew, 2014). We conclude that haptic stimulations increase the involvement level of human subjects during hand rehabilitation training. Potentially, the proposed fingertip cutaneous stimulation system can be used in rehabilitation training that can better attract user’s attention during treatment. According to Piggott et al., the benefits of using haptic devices in upper-limb rehabilitation include creating more immersive virtual reality and contributing to the recovery of sensory function (Piggott et al., 2016). Apart from the attention enhancement effect, combining exoskeleton-assisted hand motion and fingertip haptic stimulation may stimulate motor cortex and somatosensory cortex of the brain simultaneously, and thus further promote motor function recovery. Apart from the attention levels, other more direct indicators reflecting the degree of active involvement of the subjects should also be investigated in the future studies. Our future work includes further investigation of the effects of haptic stimulation on functional areas of the brain. The experiment results also showed that participants had significantly higher attention levels when the higher stimulation level (simulating grasping a 300 g glass) rather than the lower stimulation level (simulating grasping a 150 g glass) was applied (*p* = 5.515 × 10^−6^). This figure suggests that stronger haptic stimulation yields higher attention levels of the participants. But please note that too much pressure added to the fingertips by the haptic actuators may cause discomfort to the user. In this study, only a glass grasping task is involved. In the future study, other influence factors such as the types of grasping and the fingertip haptic feedback modalities will be studied in order to further understand the mechanism of the attention enhancement. What’s more, in the present experiment, only a group of young, healthy people participated. In other words, the attention enhancement effect of integrating haptic stimulation into the exoskeleton-assisted hand exercise was only proved on healthy subjects. This is one of the limitations of our current study. In future studies, a greater number of stroke patients should be included to further prove the clinical feasibility of the proposed method.

In this study, the cutaneous haptic stimulation actuators only provide normal force stimulus to the fingertips, which is perpendicular to the actuator surface. To create a more vivid haptic experience, the tangential contact force during the grasping interaction should also be provided. However, the complexity of the actuators and the difficulty of the control will be significantly increased. Moreover, the normal force is much larger than the tangential force during the grasping interaction as we observed in our experiment. There might be a trade-off between providing a more vivid haptic experience and designing the complexity of the actuators’ system. Please note that providing vivid haptic experience of grasping is not the main purpose of this study. In other words, to accurately simulate the grasping process is not the main goal of the study. It is used as a mean to enhance the attention of the user during the hand rehabilitation training process. Of course, if other haptic information like the slippery effects is added, it may provide a more vivid interaction experience for the user. Since our concept is to provide more stimulation with finger extension/flexion assistance to attract the patient’s attention during the hand rehabilitation, we argue that providing less haptic information than the actual grasping scene does not affect our purpose. In our future studies, we will try to improve the actuator structure and control algorithm to provide a more vivid interaction experience.

## Data Availability

The raw data supporting the conclusions of this article will be made available by the authors, without undue reservation.
